# Mitral annular calcification is associated with atrial fibrillation and major cardiac adverse events in atrial fibrillation patients: A systematic review and meta-analysis

**DOI:** 10.1097/MD.0000000000017548

**Published:** 2019-11-01

**Authors:** Yimin Li, Zhiping Lu, Xiangyu Li, Jin Huang, Qinghua Wu

**Affiliations:** a Department of Cardiology, Second Affiliated Hospital of Nanchang University, Nanchang; b Department of Cardiology, Affiliated Brain Hospital of Nanjing Medical University (Chest Branch), Nanjing, China.

**Keywords:** atrial fibrillation, major cardiac adverse events, meta-analysis, mitral annulus calcification

## Abstract

**Background:**

The incidence of atrial fibrillation (AF) varies from 5.4% to 47.1% in patients with mitral annulus calcification (MAC). We conducted a systematic review and meta-analysis on the association between MAC and AF, as well as the relation between MAC and major cardiac adverse events (MACEs) in AF patients.

**Methods:**

We conducted comprehensive search for literature on associations between MAC and AF using the following databases: MEDLINE, PubMed, Embase, and the Web of Science. The pooled odds ratio (OR) or relative risk and the corresponding 95% confidence intervals (CIs) were calculated to assess the relationship between MAC and AF, as well as the rates of MACEs in AF patients with or without MAC.

**Results:**

Thirteen studies met our eligibility criteria on associations between MAC and AF, including 6232 patients with MAC and 15,199 patients without MAC. Moreover, 5 studies met our eligibility criteria on the rates of MACEs in AF patients with or without MAC. The pooled analysis demonstrated a statistically significant increased risk of development of incident AF in patients with MAC than those without MAC (random effects OR: 2.34; 95% CI: 1.91, 2.85; *P* = .000). And the pooled analysis demonstrated a statistically significant increased risk of development of MACEs in AF patients with MAC (random effects OR: 2.34; 95% CI: 1.24, 4.41; *P* = .009).

**Conclusion:**

MAC was independently associated with AF and AF patients with MAC were at greater risk for cardiovascular and cerebrovascular events.

## Abstract 2

Supplemental Digital Content is available in the text

## 1 Introduction

Atrial fibrillation (AF) is the most common cardiac arrhythmia but non-fatal in itself.^[1]^ AF is governed by both clinical and genetic factors^[2]^ and its prevalence is age-related and rises significantly from 0.5–1% to 8% after 80 years of age.^[3]^ AF, associated with its complications such as stroke and myocardial infarction, generates a substantial burden in terms of costs, morbidity, and mortality. Given that patients with paroxysmal or asymptomatic AF and elderly people over 60 with other risk factors like hypertension, coronary heart disease, and diabetes may remain undiagnosed or underdiagnosed, the morbidity and the menace of AF may be underestimated.^[4]^ Therefore, further study of the disease is still particularly important.

Mitral annulus calcification (MAC) is characterized by calcium and lipid deposition in the fibrous support structure of the mitral valve.^[5]^ It is initially thought to be an age-related process. However, many reported studies showed that MAC is associated with a variety of conditions including ventricular and atrial enlargement, congestive heart failure, structural and functional impairment of the aortic and mitral valve, endocarditis, conduction defects, carotid artery stenosis, coronary artery disease, and ischemic strokes.^[6]^ MAC is a predictor of incident AF and is independently associated with all-cause mortality and with cardiovascular morbidity and mortality in patients with AF.^[7]^ O’Neal et al also found that MAC was a predictor of incident AF in 6641 participants free of clinical cardiovascular disease and AF at baseline.^[8]^ However, currently, there is scant data on the relationship between MAC and cardiovascular morbidity and mortality in AF patients. Potpara et al studied 1056 nonvalvular AF patients and found that MAC correlated with all-cause death (hazard ratios [HR], 4.3; 95% confidence interval [CI], 1.8–10.0; *P* < .001), and cardiovascular death (HR, 3.5; 95% CI, 1.2–10.4; *P* = .025).^[9]^ By contrast, Mazzone et al showed that MAC was an independent predictor of all-cause mortality in patients with sinus rhythm (HR, 1.74; 95% CI, 1.07–2.82, *P* = .02), but did not predict all-cause mortality in AF patients.^[10]^

In the present study, we conducted a systematic review and meta-analysis of observational cross-sectional, case-control, or cohort studies on MAC and AF to examine the association between MAC and AF, as well as the relation between MAC and major cardiac adverse events (MACEs) in AF patients.

## 2 Methods

### 2.1 Search strategy

This study followed the protocols specified in the preferred reporting items for systematic reviews and meta-analysis statement.^[11]^ A comprehensive search was conducted independently by 2 reviewers (YML and XYL) for literature on associations between MAC and AF using the following databases: MEDLINE, PubMed, Embase, and the Web of Science. To identify and retrieve all potentially relevant articles regarding this topic, we used the following terms in the search: ([“mitral annulus calcification” OR “mitral annular calcification” OR “mitral annulus calcium” OR “mitral annular calcium” OR “mitral valve”] AND [“atrial fibrillation” OR “auricular fibrillation” OR “persistent atrial fibrillation” OR “paroxysmal atrial fibrillation”]). A manual search was also performed by analyzing the reference list of retrieved original publications and review articles.

### 2.2 Eligibility criteria

Articles published between March 1, 1988 and March 1, 2018 were included. The current meta-analysis included observational cross-sectional, case-control, or cohort studies that allowed for assessment of associations between MAC and AF and the rates of MACEs in AF patients with or without MAC. AF was defined as electrocardiographic (ECG) recording of AF (paroxysmal, persistent, or permanent) from standard 12-lead ECG, Holter monitoring, and/or documented diagnosis of AF by International Classification of Diseases, Ninth Revision. MAC was assessed by transesophageal echocardiography or computed tomography scan. MACEs were defined as stroke, myocardial infarction, and all-cause death. Studies were excluded if they were conducted in specific populations: patients with thyroid and parathyroid diseases, chronic kidney diseases or immune diseases, and children. If multiple articles were published from the same cohort, the latest paper was chosen.

### 2.3 Data extraction

Data from relevant studies were extracted independently by 2 reviewers (YML and XYL) using a standard form. Any disagreement was discussed between the 2 reviewers or all authors to reach a consensus. The following data were extracted: title of study, name of first author, country of origin, publication year, number of participants in the MAC group and the control group, demographic data of participants, number of cases of AF, mean follow-up duration, and cases of MACEs. The status of AF includes prevalent AF (cases of AF at the time of inclusion) and incident AF (cases of developed AF in the follow-up period).

### 2.4 Data quality assessment

Newcastle–Ottawa quality assessment scale was used to evaluate each study in 3 domains: recruitment and selection of the participants, similarity and comparability between the groups, and ascertainment of the outcome of interest among cohort studies.^[12]^ The quality of cross-sectional study was assessed as described by Rostom et al.^[13]^ Furthermore, the risk of bias of the included studies was assessed with the following criterions:

(1) random sequence generation,
(2) allocation concealment,
(3) blinding of participants and personnel,
(4) blinding of outcome assessment,
(5) incomplete outcome data,
(6) selective reporting, and
(7) other biases.

### 2.5 Ethical statement

All results and analyses were from previous published studies; thus, no ethical approval and patient consent are required.

### 2.6 Statistical analysis

The pooled odds ratio (OR) or relative risk and the corresponding 95% CIs were calculated to assess the relationship between MAC and AF, as well as the rates of MACEs in AF patients with or without MAC. Heterogeneity was assessed using the Chi-square test based on *Q*- and *I*^2^- statistic. For *Q* statistic, substantial heterogeneity was defined as *P* > .10. The *I*^2^ statistic ranges from 0% to 100% (*I*^2^ **<** 25%, low heterogeneity; *I*^2^ **=** 25%–50%, moderate heterogeneity, and *I*^2^ **>** 50%, substantial heterogeneity). For studies with substantial heterogeneity (*I*^2^ **>** 50%), the random-effects model was used. If substantial heterogeneity was not present, the fixed-effects model was used to calculate the combined OR values. Otherwise, the random-effects model was used. In subgroup analysis, we compared the incidence of AF in MAC and non-MAC population between studies according to the population (community patients or inpatients) and other characteristics (study design, sample size, and gender).

We performed sensitivity analysis of included articles. All results in this analysis were considered as significant only with a 2-tailed *P* < .05. Publication biases were assessed by both Begg test and Egger test. All statistical analysis was performed by using STATA software (Version 15.0, Stata Corp, College Station, TX).

## 3 Results

### 3.1 Characteristics of the included studies

**Figure 1:**
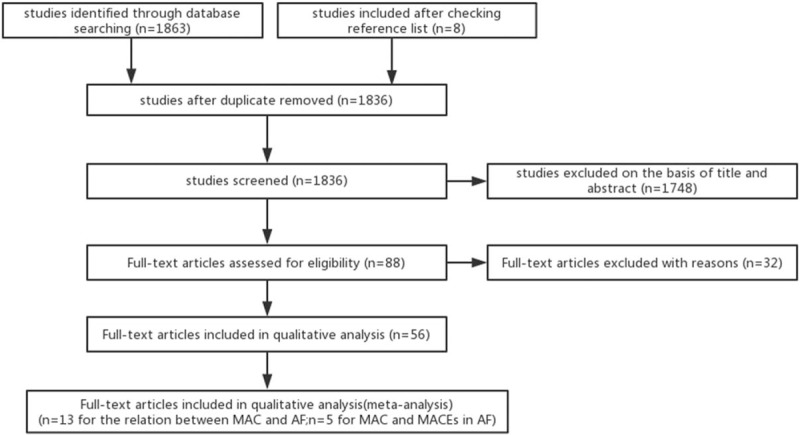
Study flowchart.

The study flowchart is shown in Figure 1. In total, our search strategy identified 1863 potentially relevant articles from initial search and 8 additional studies were further identified from the references of the retrieved articles. After exclusion of 35 duplicated articles, the titles and abstracts of 1836 articles were reviewed, and 1748 additional articles were excluded. Eighty-eight articles underwent full-text review and 32 articles were excluded because of failure to meet the eligibility requirements. Among 56 full-text articles which we reviewed, 13 studies met our eligibility criteria on associations between MAC and AF (Table 1). Among them, 9 studies are based on the cases of prevalent AF, 2 studies are based on the cases of incident AF, and another 2 studies are based on the cases of both prevalent and incident AF. These studies consisted of 21,431 individuals, including 6232 patients with MAC and 15,199 patients without MAC. In addition, the included studies were of high quality.

**Table 1:**
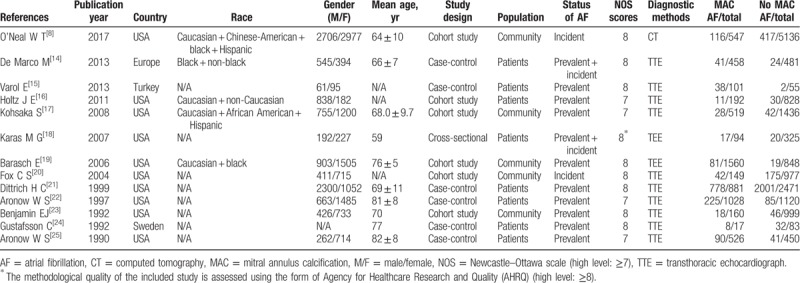
Characteristics of the included studies on association between mitral annulus calcification and atrial fibrillation.

Moreover, 5 studies met our eligibility criteria on the rates of MACEs in AF patients with or without MAC (Table 2).

**Table 2:**
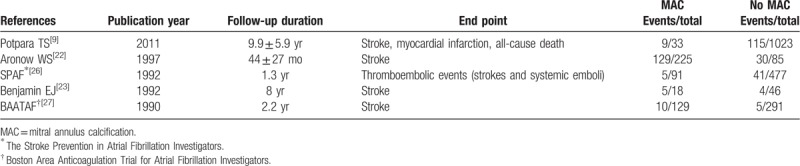
Characteristics of the included studies on the mitral annulus calcification and major adverse cardiac events in atrial fibrillation.

### 3.2 Association between MAC and AF

**Figure 2:**
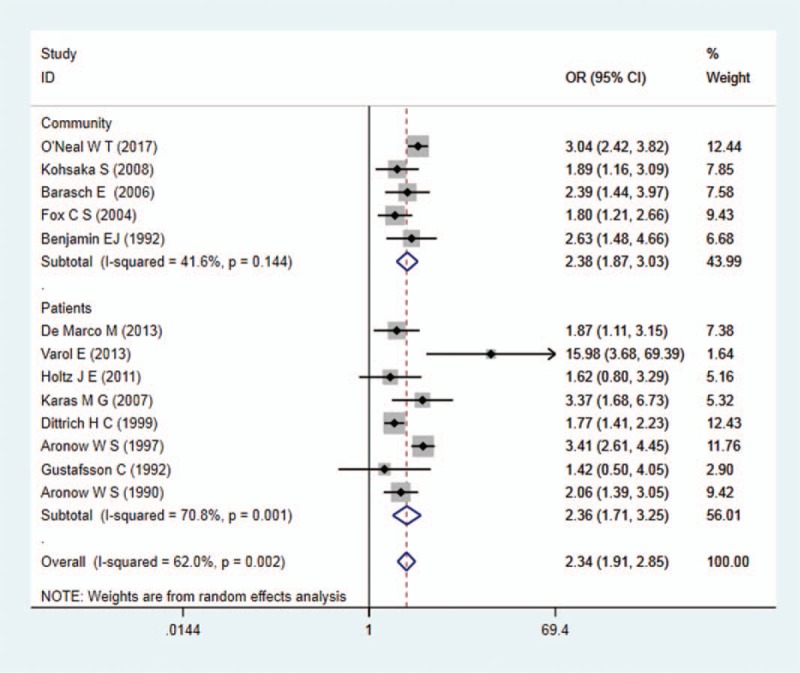
Forest plot on total studies on the relation between MAC and AF. AF = atrial fibrillation, MAC = mitral annulus calcification.

**Figure 3:**
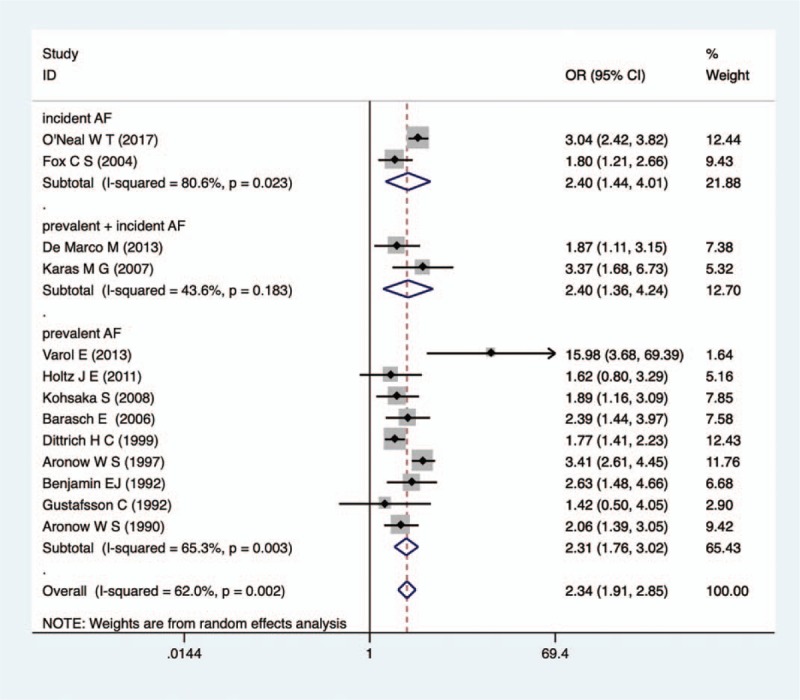
Forest plot of relation between MAC and AF according to the status of AF. AF = atrial fibrillation, MAC = mitral annulus calcification.

Twelve of the 13 studies revealed an increased risk of incident AF among patients with MAC versus those without MAC, with 10 of the 13 achieving statistical significance. The pooled analysis demonstrated a statistically significantly increased risk of development of incident AF in patients with MAC than those without MAC (random effects OR: 2.34; 95% CI: 1.91, 2.85; *P* = .000). In subgroup analysis, pooled analysis also demonstrated a statistically significantly increased risk of development of incident AF in patients with MAC than those without MAC among both community populations (random effects OR: 2.38; 95% CI: 1.97, 3.03) and inpatients (random effects OR: 2.36; 95% CI: 1.71, 3.25) (Fig. 2). The same results were obtained from subgroups according to the AF's status: AF, regardless of prevalent AF (OR: 2.34, 95% CI: 1.91, 2.85), incident AF (OR: 2.40, 95% CI: 1.44, 4.01) or both of them (OR: 2.40, 95% CI: 1.36, 4.24), is predominant in patients with MAC completely (Fig. 3).

In addition, the presence of MAC strongly favored the development of incident AF regardless of gender (males < females: OR: 2.62, 95% CI: 2.11, 3.27; males > females: OR: 1.78, 95% CI: 1.45, 2.17), sample size (n > 1000: OR: 2.31, 95%CI: 1.84, 2.91; n < 1000: OR: 2.52, 95% CI: 1.55, 4.10), or study design (Cohort: OR: 2.30, 95% CI: 1.82, 2.90; case-control: OR: 2.37, 95% CI: 1.62, 3.47) (the random effects model in all) (Supplementary Figs. 1–3). These findings strongly suggested a strong association of MAC with AF.

### 3.3 Association between MAC and MACEs in AF

**Figure 4:**
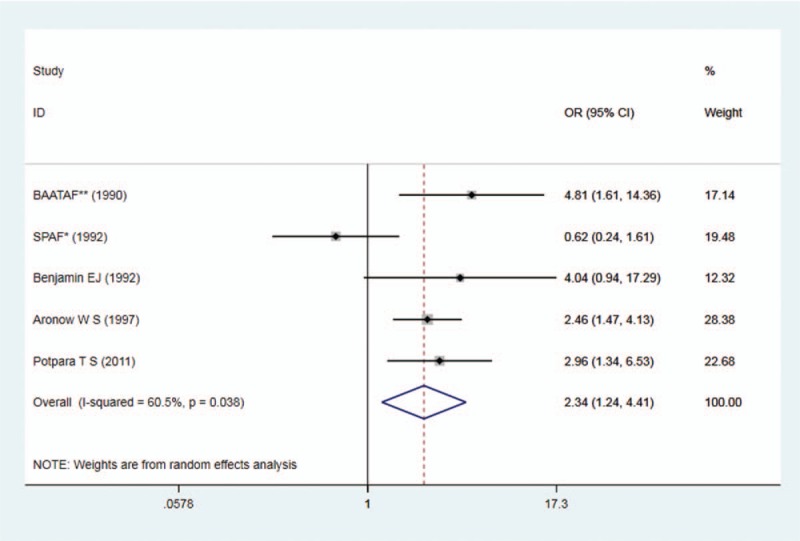
Forest plot on studies on MAC and MACEs in AF. AF = atrial fibrillation, MAC = mitral annulus calcification, MACE = major adverse cardiac events.

Totally 353 cases developed MACEs among 2418 patients with AF during median follow-up duration of at least 1.3 years. The endpoint was stroke in 3 studies, thromboembolic events (strokes and systemic emboli) in 1 study and MACEs in 1 study. Four of the 5 studies revealed an increased risk of MACEs among AF patients with MAC versus those without MAC, with 3 of the 5 achieving statistical significance. The pooled analysis demonstrated a statistically significantly increased risk of development of MACEs in AF patients with MAC (random effects OR: 2.34; 95% CI: 1.24, 4.41; *P* = .009) (Fig. 4).

### 3.4 Heterogeneity and public bias

**Figure 5:**
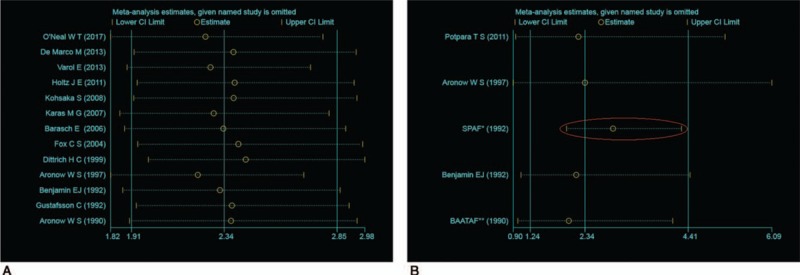
Sensitivity analysis of the relation between MAC and AF (A) and on MAC and MACEs in AF (B). AF = atrial fibrillation, MAC = mitral annulus calcification, MACE = major adverse cardiac events.

Severe heterogeneity was present in the included studies on association between MAC and AF (*I*^*2*^ = 62.0%, *P* = .002). Significant heterogeneity was still present among the community and patient population (*I*^*2*^ = 41.6%, *P* = .144 vs *I*^*2*^ = 70.8%, *P* = .001). Obvious heterogeneity was also found in studies on MAC and MACEs in AF patients (*I*^*2*^ = 60.5%, *P* = .038). Sensitivity analysis revealed that the pooled ORs were stable in association between MAC and AF, while the pooled ORs on MAC and MACEs in AF patients were unstable because of the SPAF study^[26]^ (Fig. 5).

**Figure 6:**
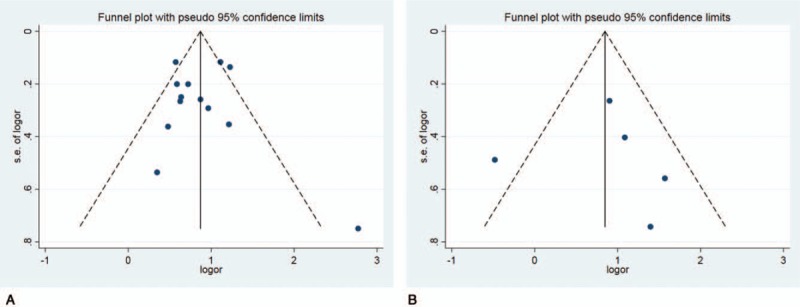
Funnel plot (A. the relation between MAC and AF, Egger test: 0.998; Begg test: 0.669; B. the MAC and MACEs in AF, Egger test: 0.939; Begg test: 0.462). AF = atrial fibrillation, MAC = mitral annulus calcification, MACE = major adverse cardiac events.

No publication bias was found through visual inspection of the funnel plot, and the result was supported by both Egger’ test and Begg’ test (the relation between MAC and AF: Egger test: 0.998; Begg test: 0.669 and the MAC and MACEs in AF: Egger test: 0.939; Begg test: 0.462) (Fig. 6).

## 4 Discussion

This systematic review and meta-analysis of 16 studies, comprising 23,958 subjects, demonstrate a significant association between MAC and AF and between MAC and MACEs in AF patients. Our subgroup analysis has further revealed that the association remains between MAC and AF regardless gender, care settings, study population size, study design, or the status of AF. Our findings also showed that AF patients with MAC have greater cardiovascular and cerebrovascular risk. Consequently, MAC may be a marker of atherosclerotic burden and increased burden for AF as well. The presence of MAC may play an important role in the understanding, prevention, and treatment of AF and MACEs in AF patients.

MAC is a common echocardiographic finding in 9% of women and 3% of men older than 60 years.^[27]^ In 2 population-based studies from the cohort of the Framingham Heart Study^[28]^ and the Multi-Ethnic Study of Atherosclerosis,^[29]^ MAC is proved to be closely associated with AF. Meanwhile, MAC and AF interact in many ways. MAC was associated with increased cardiovascular morbidity, cardiovascular mortality, and all-cause mortality of AF patients in the Belgrade Atrial Fibrillation Study.^[9]^ In the Framingham study, MAC conferred a greater risk for incident stroke than AF did and there was a continuous relation between severity of MAC and stroke risk.^[14]^ MAC is also a predictor of the recurrence of paroxysmal AF after cryothermal ablation.^[30]^ Both MAC and AF are highly prevalent in patients undergoing transcatheter aortic valve implantation (TAVI), and severe MAC is associated with increased all-cause and cardiovascular mortality.^[31,32]^ Hence, MAC may not only be a risk factor for AF, but may also be an important prognostic predictor.

Left atrial enlargement is regarded as a key medium to the association between MAC and AF in the Strong Heart Study.^[33]^ MAC can cause mild or moderate mitral regurgitation,^[7]^ which will result in volume and pressure overload in the left atrium, leading to left atrial enlargement and predisposing subjects to AF.^[20]^ MAC may also interrupt inter-atrial and intra-atrial conduction, leading to conduction system and atrial conduction defects, thus resulting in AF.^[34]^ Finally, MAC may be associated with other unappreciated risk factors, such as arterial stiffness or inflammation that could predispose patients to the development of AF.^[35]^

MAC is also reported to be related to more cardiovascular events.^[19,36]^ In these reports; however, MAC is considered a risk factor for coronary atherosclerosis, leading to adverse events.^[9]^ In patients with AF, the relationship between MAC and cerebrovascular diseases is still controversial. Randomized controlled studies also showed increased stroke risk in patients with MAC; AF was observed in Boston Area Anticoagulation Trial for Atrial Fibrillation Investigators ^[27]^ while it was not significant in the stroke prevention in atrial fibrillation trial (SPAF).^[26]^ In our study, MAC in AF patients was directly related to cardiovascular and cerebrovascular adverse events. It is noteworthy that MAC itself could serve as a nidus for thrombus formation.^[23]^

These findings of our study have important clinical significance. MAC not only predicts AF development but its presence infers an increased risk of adverse cardiovascular outcomes among those who already have the arrhythmia.^[8]^ For patients with MAC, clinicians should take measures to prevent AF and to reduce the burden of adverse outcomes if AF is apparent. MAC patients prone to AF may need more reasonable rhythm control strategies, while patients already with complicated AF may need more rigorous anticoagulation regimens. Additionally, considering the prognostic value of MAC in AF ablation^[30]^ and TAVI,^[32]^ MAC is a potential preoperative and post-operative evaluative factor.

Meanwhile, our study has several limitations. First and foremost, we observed substantial heterogeneity in the included studies. The clinical features of the population may be the main reason for the heterogeneity. For example, community populations or patients, different ages, different races, and other factors may all contribute to heterogeneity. Only 5 studies conducted research in the community population, and the age span was also large, from 17 to 103 years. Subgroup analysis based on community populations showed that AF was highly prevalent in the MAC population and heterogeneity declined sharply. Meanwhile, through sensitivity analysis, we found that the pooled ORs were not stable because the large sample size and the high prevalence of AF (78.2%) in the study by Dittrich et al,^[21]^ which may explain the instability of the results. According to sensitivity analysis on studies on MAC and MACEs in AF, the study by SPAF^[26]^ was the key contributor to the instability of the pooled ORs. As it met the eligibility criteria, we did not remove this study. In addition, as this study was a study-level meta-analysis, due to the lack of patient-level data, inherent clinical heterogeneity among trials should be taken into consideration in the interpretation of our findings. Moreover, due to limited data, further stratified analysis was unavailable. Therefore, the study of MAC and AF still needs further research in subgroups of different individuals and age groups, and also requires dynamic observation in single population.

In conclusion, the present article indicates that MAC is independent predictor of AF patients. A general consideration emerges from this analysis concerning the identification of MAC. MAC is often considered a trivial finding and is not worth reporting. Our analysis strongly suggests that this is not the case and that evidence of MAC should be highlighted, especially in patients with AF. According to the severity of MAC, it is particularly important to further explore risk stratification of AF by MAC.

## Author contributions

**Conceptualization:** Yimin Li.

**Data curation:** Yimin Li, Xiangyu Li.

**Formal analysis:** Yimin Li.

**Methodology:** Zhiping Lu.

**Project administration:** Zhiping Lu.

**Resources:** Zhiping Lu.

**Software:** Zhiping Lu, Xiangyu Li.

**Validation:** Xiangyu Li.

**Visualization:** Xiangyu Li.

**Writing – original draft:** Yimin Li.

**Writing – review and editing:** Jin Huang, Qinghua Wu.

## Supplementary Material

Supplemental Digital Content
