# Supplementary figures and images for: Mitral annular calcification is associated with atrial fibrillation and major cardiac adverse events in atrial fibrillation patients: A systematic review and meta-analysis
Source: Medicine (Baltimore). 2019 Nov 1;98(44):e17548. doi: 10.1097/MD.0000000000017548 (PMC6946188; doi:10.1097/MD.0000000000017548)

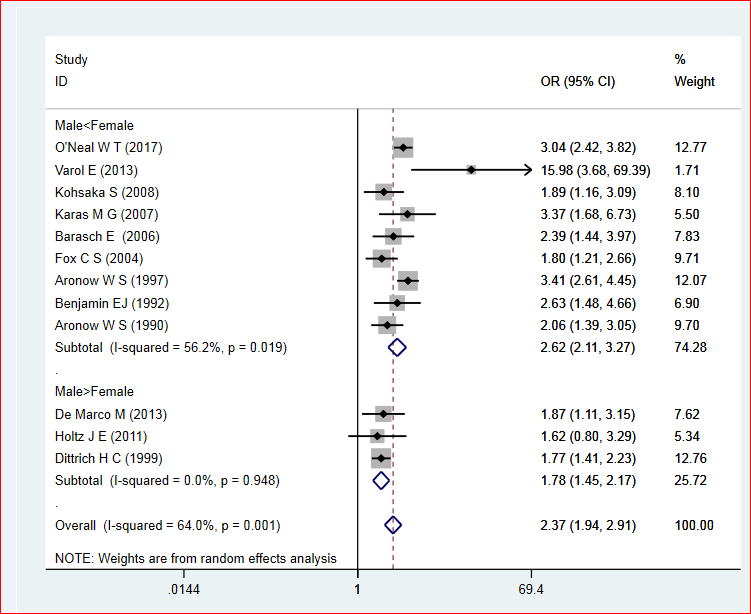


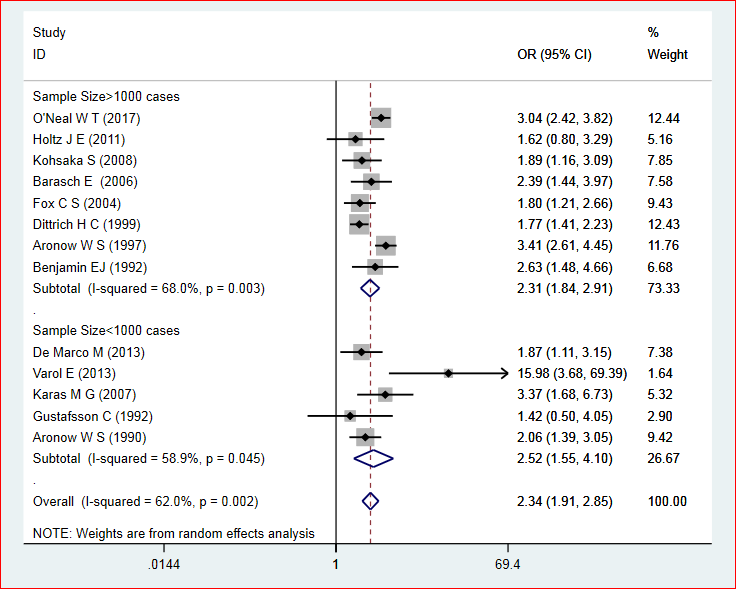


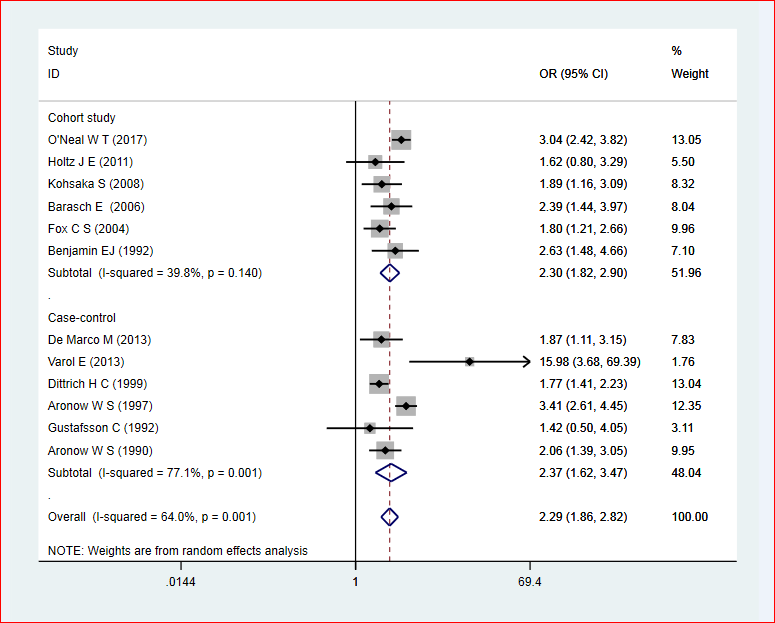

Supplement: Supplemental Digital Content [file medi-98-e17548-s001.doc]
